# Prevalence and factors associated with post-stroke depression: a cross-sectional analysis of a prospectively assembled cohort in Eastern Saudi Arabia

**DOI:** 10.3389/fneur.2026.1863484

**Published:** 2026-08-26

**Authors:** Aishah Albakr, Mustafa Alqarni, Ali Alhashim, Dalal Albakr, Zakia Yasawy, Omar Al Ghamdi, Erum Sharif, Rizwana Shahid, Azra Zafar, Saima Nazish, Wa’ad Massoud Almunassir Alqahtani, Dana Aljamea, Fahad Aldamigh, Fahad AlDawsari, Ziyad Al Ghannam

**Affiliations:** 1 Department of Neurology, College of Medicine, Imam Abdulrahman Bin Faisal University, Dammam, Saudi Arabia; 2 Department of Psychiatry, College of Medicine, Imam Abdulrahman Bin Faisal University, Dammam, Saudi Arabia

**Keywords:** depression screening, functional disability, neurological severity, NIHSS (National Institute of Health Stroke Scale), post-stroke depression, Saudi Arabia

## Abstract

**Objective:**

To estimate the prevalence and severity of post-stroke depression (PSD) and identify factors independently associated with PSD among stroke survivors.

**Methods:**

This cross-sectional analysis was conducted within a prospectively assembled hospital-based cohort at King Fahd University Hospital between January 2021 and November 2025. Adults with ischemic stroke, transient ischemic attack, intracerebral hemorrhage, or cerebral venous sinus thrombosis (CVST) underwent PSD assessment at least 3 months after the index event. PSD was established through structured clinical assessment based on DSM-5-TR and ICD-11 criteria together with the clinician-administered 17-item Hamilton Depression Rating Scale. Factors associated with PSD were evaluated using forced-entry multivariable logistic regression.

**Results:**

Among 403 participants, the mean age was 57.3 ± 13.8 years, and 69.2% were male. PSD was identified in 172 participants (42.7%): 71 (17.6%) had mild, 61 (15.1%) moderate, and 40 (9.9%) severe or very severe depression. The complete-case multivariable analysis included 398 participants. Higher NIHSS score was independently associated with PSD [adjusted odds ratio (aOR) per 1-point increase, 1.35; 95% CI, 1.22–1.49; *p* < 0.001], as was unfavorable functional status at discharge (mRS 3–5 vs. 0–2: aOR, 6.51; 95% CI, 3.19–13.29; *p* < 0.001). The overall stroke-subtype test was nonsignificant (*p* = 0.067), although the category-specific CVST coefficient indicated higher odds of PSD (aOR, 5.73; 95% CI, 1.34–24.50; *p* = 0.018). The model demonstrated excellent apparent discrimination (AUC, 0.89; bootstrap 95% CI, 0.86–0.92), with 72.5% sensitivity and 90.5% specificity at a cutoff of 0.50.

**Conclusion:**

PSD affected approximately two-fifths of participants and was most consistently associated with neurological severity and functional disability. Standardized depression screening should be integrated into post-stroke care, particularly for patients with greater neurological impairment or functional dependence. The CVST finding was exploratory and requires confirmation.

## Introduction

Stroke is a major neurovascular disorder that frequently results in persistent physical, cognitive, and emotional impairment (1, 2). Each year, approximately 15 million people worldwide experience a stroke. Post-stroke depression (PSD) is among the most common neuropsychiatric complications following stroke, affecting approximately one-third of survivors, although reported prevalence varies according to study population, diagnostic criteria, assessment methods, and timing of evaluation (3–7).

Multiple clinical and demographic features have been identified as major risk factors for the development of PSD, including increased stroke severity, greater functional disability, cognitive impairment, and a history of depression (5, 6, 8, 9). The pathophysiology of PSD is multifactorial and remains incompletely understood. Proposed biological mechanisms include disruption of fronto-limbic and mood-regulating neural networks, neuroinflammatory activation, and alterations in monoaminergic neurotransmission (9–11). Psychological stress, loss of functional independence, reduced social participation, and sociocultural factors may also contribute to its development and persistence (12–16).

Stroke and depression share a complex and bidirectional relationship. Strokes heighten vulnerability to depressive symptoms, whereas underlying depression is recognized as an independent risk factor for subsequent stroke and inadequate recovery (9, 17–20). Patients with PSD commonly demonstrate slower motor improvement, reduced participation in rehabilitation therapy, impaired activities of daily living, diminished quality of life, and greater dependency. Importantly, PSD has also been associated with increased mortality after stroke, underscoring its clinical significance (10, 19–24). Encouragingly, timely detection and effective management of PSD—through pharmacological, psychological, and structured psychosocial interventions—can enhance functional recovery and promote greater independence (11, 25, 26).

In Saudi Arabia and the wider Middle East, stigma surrounding mental illness, culturally shaped beliefs about psychological disorders, and limited recognition of depressive symptoms may discourage formal help-seeking, contributing to delayed diagnosis and reduced access to appropriate mental health services (9, 27–29). The implementation of standardized screening instruments, such as the Hospital Anxiety and Depression Scale (HADS), has demonstrated feasibility and effectiveness for identifying depression among stroke survivors in the region and may facilitate earlier recognition and intervention (10, 29–31).

Recognition of PSD in clinical practice is often challenging, specifically when patient-reported distress or disability appears out of proportion to the post-stroke neurological deficits. Diagnostic reliability might be further limited by stroke-related factors, including cognitive impairment, aphasia, and impaired emotional insight (21, 32, 33). A recent systematic review identified meaningful differences in the phenomenology of post-stroke depression compared with primary depressive disorders, highlighting the importance of stroke-specific diagnostic frameworks that account for neurological deficits and stroke-related cognitive impairment (34).

Evidence regarding clinician-confirmed PSD and its independent clinical correlates remains limited in Saudi Arabia and the wider Middle East. The present study was designed to address this gap by evaluating PSD through structured clinical assessment in a relatively large hospital-based cohort encompassing multiple cerebrovascular-event types and by examining the relative contributions of neurological severity, functional disability, and stroke-related characteristics.

Accordingly, this study aimed to estimate the prevalence and severity of PSD and to identify sociodemographic, neurological, functional, and stroke-related factors independently associated with PSD. A clearer understanding of these factors may support early recognition, timely intervention, and the integration of mental health assessment into post-stroke care.

## Materials and methods

### Study design and participants

This study represents a cross-sectional analysis of post-stroke depression conducted within a prospectively assembled hospital-based cohort. Consecutive adults (≥18 years) evaluated at King Fahd University Hospital for a suspected acute cerebrovascular event between January 2021 and November 2025 were screened for eligibility. Patients with confirmed acute ischemic stroke (AIS), transient ischemic attack (TIA), intracerebral hemorrhage (ICH), or cerebral venous sinus thrombosis (CVST) who met the eligibility criteria were prospectively enrolled.

The original study protocol was approved by the Standing Committee for Research Ethics on Living Creatures at Imam Abdulrahman Bin Faisal University (approval No. 2017-200-Med-NF). A revised protocol was subsequently approved by the Institutional Review Board on February 16, 2023 (IRB No. IRB-2023-01-078). The protocol was further amended and extended under the same IRB number on April 30, 2026. Written informed consent was obtained from all participants or their legally authorized representatives before enrollment. The study was conducted in accordance with the Declaration of Helsinki, and reporting followed the Strengthening the Reporting of Observational Studies in Epidemiology (STROBE) guidelines (35).

Cerebrovascular-event diagnoses were established according to World Health Organization criteria and confirmed by computed tomography and/or magnetic resonance imaging (36). All eligible cerebrovascular-event types and etiological categories were considered for inclusion. For the present cross-sectional analysis, PSD was assessed once at the first eligible follow-up visit occurring at least 3 months after the index cerebrovascular event.

Adequate language comprehension was required to ensure reliable psychiatric assessment and was evaluated using the Frenchay Aphasia Screening Test (FAST), together with clinical judgment. Participants with severe aphasia that precluded a valid clinical interview or reliable completion of the depression assessment were excluded (37). Participants were also required to have sufficient cognitive function to complete a valid clinical interview and symptom assessment. Cognitive screening was performed using the Montreal Cognitive Assessment (MoCA). Participants with severe cognitive impairment precluding reliable psychiatric assessment, defined by a MoCA score <10 together with clinical judgment, were excluded (38).

Additional exclusion criteria included nonvascular causes of brain injury, a documented history of major depressive disorder or another psychiatric disorder, dementia, pre-stroke antidepressant use, incomplete data required to establish the primary outcome, or failure to complete the scheduled follow-up assessment.

### Data collection and timing

Data were obtained from a prospectively maintained comprehensive stroke registry at King Fahd University Hospital. The registry captured standardized demographic, socioeconomic, vascular, neurological, imaging, treatment, and functional data from the index cerebrovascular hospitalization and subsequent outpatient follow-up visits. Data were entered and verified by trained neurology residents and members of the stroke team using participant or caregiver interviews, electronic medical records, stroke-service documentation, neuroimaging reports, and direct clinical assessments.

Demographic and socioeconomic variables included age, sex, marital status, educational attainment, employment status, and socioeconomic status. Vascular risk factors included hypertension, diabetes mellitus, dyslipidemia, current smoking, atrial fibrillation, and previous stroke or TIA.

Index-event variables included cerebrovascular-event type, ischemic-stroke etiology, lesion laterality, vascular territory, cortical or subcortical involvement, and receipt of hyperacute reperfusion therapy. Hyperacute reperfusion therapy comprised intravenous thrombolysis, endovascular thrombectomy, or both. Stroke subtype, etiology, and anatomical classifications were determined by the treating stroke team using the clinical presentation, diagnostic investigations, and available neuroimaging findings.

Clinical neurological manifestations recorded in the registry included motor weakness, sensory symptoms or pain, speech or language impairment, visual disturbance, neglect, mild cognitive impairment, vertigo or imbalance, dysphagia, and urinary incontinence. Neurological severity was assessed at hospital discharge using the National Institutes of Health Stroke Scale (NIHSS), and functional status was assessed at discharge using the modified Rankin Scale (mRS) (39, 40).

Follow-up data were collected during scheduled visits to the neurology outpatient clinic. The timing and procedures used for PSD assessment are described below.

### Assessment of post-stroke depression

PSD was assessed once at the first eligible outpatient follow-up visit occurring at least 3 months after the index cerebrovascular event. Assessment comprised a structured face-to-face clinical interview combined with administration of the 17-item Hamilton Depression Rating Scale (HDRS-17) (31, 41). Interviews and HDRS-17 assessments were conducted by trained neurology residents and subsequently reviewed and confirmed by a stroke neurology consultant. Psychiatric consultation was obtained for diagnostically complex or uncertain cases.

PSD was defined by clinically significant depressive symptoms consistent with the Diagnostic and Statistical Manual of Mental Disorders, Fifth Edition, Text Revision (DSM-5-TR), and the International Classification of Diseases, 11th Revision (ICD-11), together with an HDRS-17 score ≥8 (41–43). HDRS-17 scores were used to classify depression severity as mild (8–13), moderate (14–18), severe (19–22), or very severe (≥23) (31, 41).

To improve diagnostic specificity, PSD was differentiated from anxiety disorders, post-stroke post-traumatic stress disorder (PTSD), and acute stress reactions. The Frenchay Aphasia Screening Test (FAST), Montreal Cognitive Assessment (MoCA), and Generalized Anxiety Disorder 7-item scale (GAD-7) were used to evaluate language comprehension, cognitive function, and anxiety symptoms, respectively (37, 38, 44). Findings from these standardized assessments were integrated with the clinical interview to establish the final diagnosis. Participants with anxiety symptoms, PTSD, or acute stress reactions were not excluded solely on the basis of these post-stroke manifestations. Those who completed a valid assessment but did not meet the diagnostic criteria for PSD were classified in the no-PSD group.

Although all clinical diagnoses and HDRS-17 assessments were reviewed by a stroke neurology consultant, formal inter-rater reliability was not evaluated. Assessors were not formally blinded to neurological severity or functional status.

The primary outcome was clinician-confirmed PSD at the assessment performed at least 3 months after the index cerebrovascular event.

### Statistical analysis

Statistical analyses were performed using IBM SPSS Statistics version 25 (IBM Corp., Armonk, NY, USA). Continuous-variable distributions were assessed through visual inspection of histograms and the Shapiro–Wilk test. Normally distributed continuous variables are presented as mean ± standard deviation (SD), whereas non-normally distributed variables are presented as median and interquartile range (IQR), unless otherwise specified for descriptive purposes. Categorical variables are presented as frequencies and percentages.

Participant characteristics were compared between those with and without PSD. Categorical variables were compared using the Pearson chi-square test or Fisher’s exact test when expected cell counts were small. Continuous variables were compared using the independent-samples *t* test for normally distributed variables and the Mann–Whitney *U* test for non-normally distributed variables. Effect sizes were reported using Cohen’s *d* for normally distributed continuous variables, the Phi coefficient for binary categorical comparisons, and Cramér’s *V* for categorical variables with more than two categories.

Additional exploratory analyses examined stroke laterality, vascular territory, and less frequent neurological manifestations. Analyses of stroke laterality and vascular territory were restricted to participants with ischemic stroke or intracerebral hemorrhage for whom these anatomical classifications were applicable. Fisher’s exact or Fisher–Freeman–Halton exact tests were used when expected cell counts were small.

Variables associated with PSD at *p* < 0.10 in univariable analyses, together with clinically relevant covariates selected on the basis of previous evidence, were considered for multivariable analysis. The final set comprised 13 predictor domains: NIHSS score, functional disability, stroke subtype, cortical involvement, dysphagia, hyperacute reperfusion therapy, sex, employment status, urinary incontinence, mild cognitive impairment, speech or language deficit, motor weakness, and sensory symptoms. These predictor domains were entered simultaneously into a multivariable logistic regression model using the forced-entry method.

PSD was coded as the event of interest (PSD = 1; no PSD = 0); therefore, an adjusted odds ratio (aOR) greater than 1 indicates increased adjusted odds of PSD. NIHSS score was entered as a continuous variable, with its aOR reported per 1-point increase. Functional disability at discharge was dichotomized as mRS 0–2 versus 3–5, representing favorable and unfavorable functional status, respectively. Stroke subtype was entered as a categorical predictor, with ischemic stroke as the reference category and separate coefficients estimated for ICH, CVST, and TIA. Employment status was also entered categorically, with employment as the reference category and separate coefficients estimated for unemployment, housewife status, and retirement. Overall *p* values for stroke subtype and employment status were obtained using joint Wald tests. Adjusted odds ratios with 95% confidence intervals (CIs) were reported.

Multicollinearity was assessed using variance inflation factors (VIFs) and tolerance statistics. Model significance was evaluated using the likelihood-ratio test, model fit was assessed using the Hosmer–Lemeshow goodness-of-fit test, and discrimination was evaluated using the area under the receiver operating characteristic curve (AUC). The apparent 95% CI for the AUC and the pointwise 95% confidence band for the ROC curve were estimated using 5,000 stratified bootstrap samples. Sensitivity, specificity, positive predictive value, negative predictive value, and overall classification accuracy were calculated using a probability cutoff of 0.50.

Missing data were minimal (<2%). Available-case analysis was used for univariable comparisons when data were unavailable or a classification was not applicable. The multivariable regression included 398 participants, comprising 167 participants with PSD and 231 without PSD. Five participants with PSD were excluded from the regression because they could not be assigned to the predefined mutually exclusive model categories: one had a mixed ischemic-stroke and ICH classification, and four had combined cortical and subcortical involvement. Stroke subtype was analyzed among the remaining participants as a categorical predictor, with ischemic stroke as the reference category and separate coefficients estimated for ICH, CVST, and TIA. The model contained 17 regression coefficients, excluding the intercept, corresponding to approximately 9.8 PSD events per coefficient. All statistical tests were two-sided, and *p* < 0.05 was considered statistically significant.

## Results

### Flow of participants

**Figure 1:**
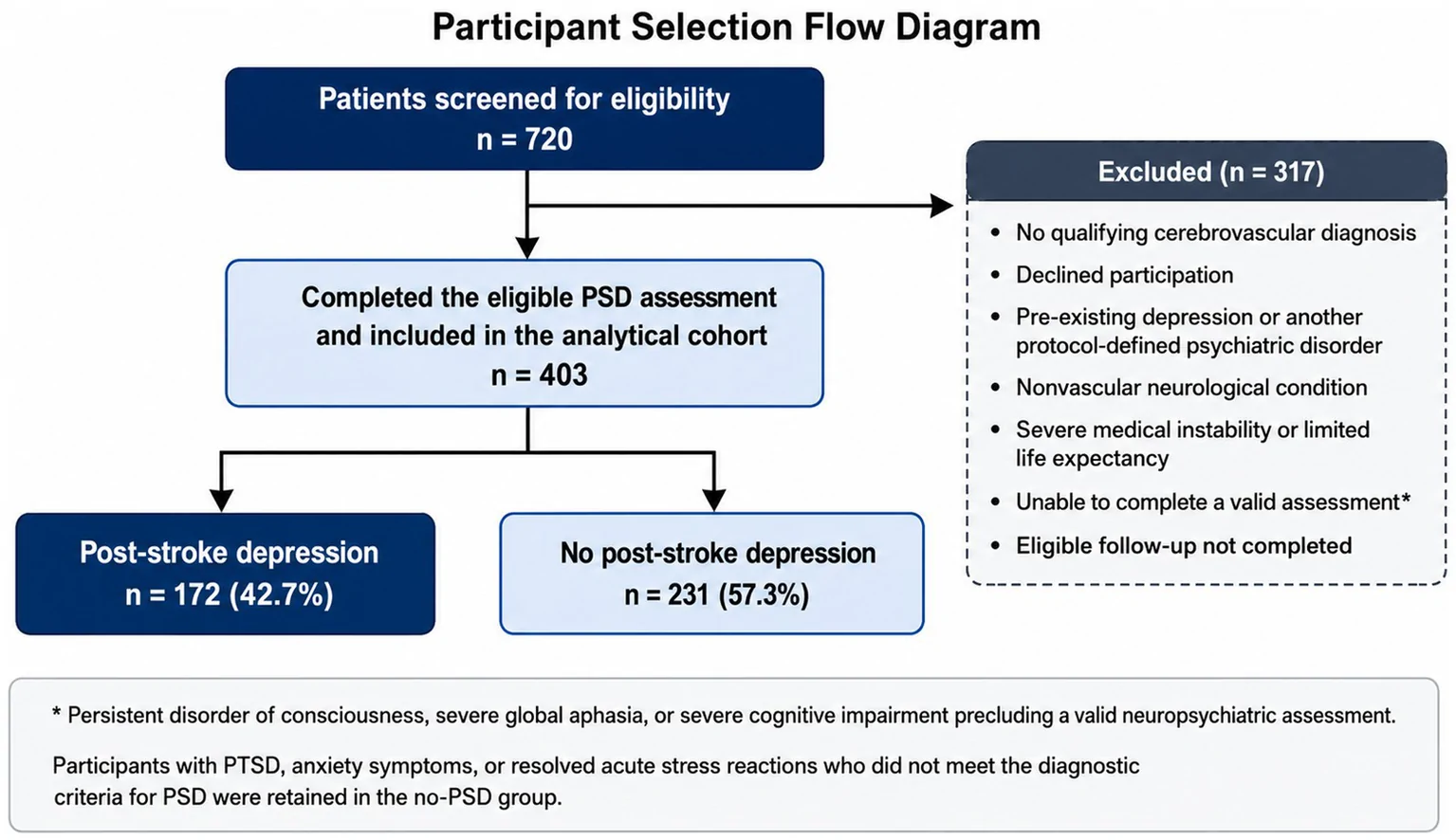
STROBE flow diagram illustrating participant selection. Of 720 patients screened for eligibility, 317 were excluded because they did not have a qualifying cerebrovascular diagnosis, met one or more exclusion criteria, declined participation, or did not complete the eligible follow-up assessment. The final analytical cohort comprised 403 participants: 172 with post-stroke depression (PSD) and 231 without PSD. Participants with anxiety symptoms, post-stroke post-traumatic stress disorder, or resolved acute stress reactions who completed a valid assessment but did not meet the diagnostic criteria for PSD were retained in the no-PSD group.

A total of 720 patients were screened for eligibility. Of these, 317 were excluded because they did not have a qualifying cerebrovascular diagnosis, met one or more exclusion criteria, declined participation, or did not complete the eligible follow-up assessment. The final analytical cohort comprised 403 participants who completed the PSD assessment, of whom 172 met the diagnostic criteria for PSD and 231 did not (Figure 1).

### Sample characteristics

A total of 403 participants with eligible cerebrovascular events were included. Age data were available for 402 participants, with a mean age of 57.3 ± 13.8 years. The cohort included 279 men (69.2%), and 314 participants (77.9%) were married. Employment was the largest occupational category, comprising 184 participants (45.7%). Socioeconomic status was reported as good by 165 participants (40.9%), average by 161 (40.0%), and poor by 77 (19.1%) (Table 1).

**Table 1:** Demographic and socioeconomic characteristics according to post-stroke depression status.

Characteristic	Overall (*n* = 403)	PSD (*n* = 172)	No PSD (*n* = 231)	*p* value	Effect size
Age, years, mean ± SD^1^	57.3 ± 13.8	57.8 ± 14.6	56.9 ± 13.2	0.499	*d* = 0.068
Sex				0.123	*Φ* = 0.077
Male	279 (69.2)	112 (65.1)	167 (72.3)		
Female	124 (30.8)	60 (34.9)	64 (27.7)		
Employment status				0.006	*V* = 0.175
Employed	184 (45.7)	62 (36.0)	122 (52.8)		
Unemployed	63 (15.6)	29 (16.9)	34 (14.7)		
Housewife	90 (22.3)	49 (28.5)	41 (17.7)		
Retired	66 (16.4)	32 (18.6)	34 (14.7)		
Education ≥12 years	153 (38.0)	61 (35.5)	92 (39.8)	0.372	*Φ* = 0.044
Marital status				0.237	*V* = 0.103
Married	314 (77.9)	127 (73.8)	187 (81.0)		
Single	32 (7.9)	18 (10.5)	14 (6.1)		
Widowed	46 (11.4)	23 (13.4)	23 (10.0)		
Divorced	11 (2.7)	4 (2.3)	7 (3.0)		
Socioeconomic status				0.460	*V* = 0.062
Good	165 (40.9)	69 (40.1)	96 (41.6)		
Average	161 (40.0)	74 (43.0)	87 (37.7)		
Poor	77 (19.1)	29 (16.9)	48 (20.8)		
Vascular risk factors					
Hypertension	283 (70.2)	124 (72.1)	159 (68.8)	0.479	*Φ* = 0.035
Diabetes mellitus	152 (37.7)	65 (37.8)	87 (37.7)	0.979	*Φ* = 0.001
Dyslipidemia	190 (47.1)	87 (50.6)	103 (44.6)	0.233	*Φ* = 0.059
Current smoking	80 (19.9)	38 (22.1)	42 (18.2)	0.330	*Φ* = 0.048
Atrial fibrillation	31 (7.7)	17 (9.9)	14 (6.1)	0.154	*Φ* = 0.071
Previous stroke or TIA	57 (14.1)	26 (15.1)	31 (13.4)	0.629	*Φ* = 0.024

Values are presented as *n* (%) unless otherwise indicated. ^1^indicates that age data were available for 402 participants (171 with PSD and 231 without PSD). Data for the remaining characteristics were available for all 403 participants. Age was compared using the independent-samples *t* test. Categorical variables were compared using Pearson’s chi-square test. Effect sizes are reported as Cohen’s *d* for age, the Phi coefficient (*Φ*) for binary variables, and Cramér’s *V* for variables with more than two categories. Vascular risk-factor data were available for all 403 participants.

PSD, post-stroke depression; SD, standard deviation.

### Prevalence and severity of post-stroke depression

**Figure 2:**
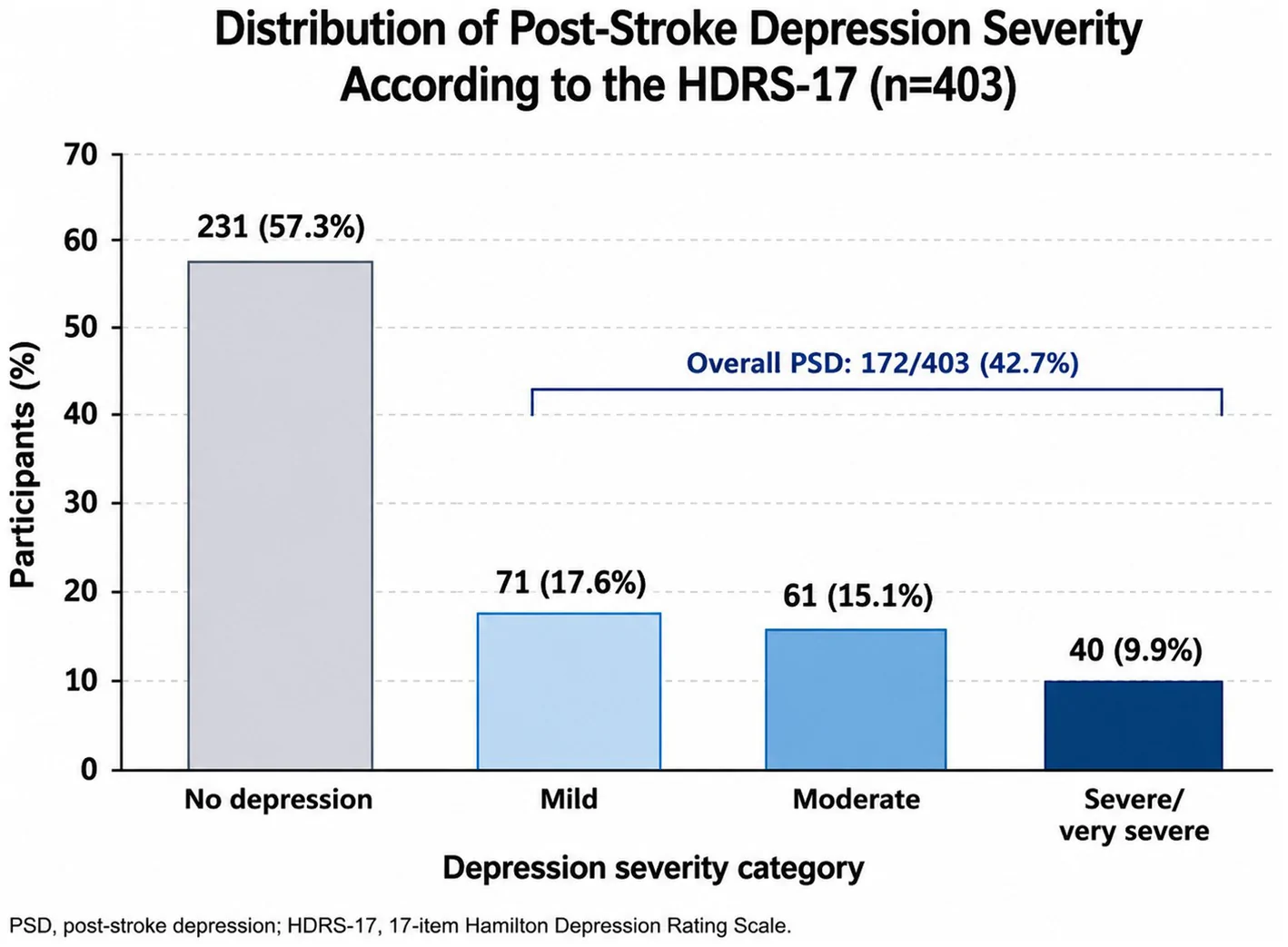
Distribution of post-stroke depression severity among the study cohort (*n* = 403) according to the 17-item Hamilton Depression Rating Scale (HDRS-17). Overall, 172 participants (42.7%) met the diagnostic criteria for PSD, including 71 (17.6%) with mild, 61 (15.1%) with moderate, and 40 (9.9%) with severe or very severe depression. The remaining 231 participants (57.3%) had no depression. PSD, post-stroke depression.

The mean HDRS-17 score was 7.66 ± 6.79. Overall, 172 of 403 participants (42.7%) met the diagnostic criteria for PSD, whereas 231 (57.3%) did not. Among the entire cohort, 71 participants (17.6%) had mild depression, 61 (15.1%) had moderate depression, and 40 (9.9%) had severe or very severe depression. The distribution of depression severity is summarized in Table 2 and illustrated in Figure 2.

**Table 2:** Distribution and severity of post-stroke depression according to the HDRS-17 (*n* = 403).

Depression category	*n* (%)
No depression	231 (57.3)
Mild depression	71 (17.6)
Moderate depression	61 (15.1)
Severe or very severe depression	40 (9.9)
Overall PSD prevalence	172 (42.7)

PSD was established through structured clinical assessment based on DSM-5-TR and ICD-11 diagnostic criteria together with an HDRS-17 score ≥8. Depression severity was classified according to HDRS-17 scores as no depression (0–7), mild (8–13), moderate (14–18), severe (19–22), or very severe (≥23). Severe and very severe depression were combined for presentation. HDRS-17 data were available for all 403 participants. Percentages were calculated using the entire cohort as the denominator and may not sum exactly because of rounding.

DSM-5-TR, Diagnostic and Statistical Manual of Mental Disorders, Fifth Edition, Text Revision; HDRS-17, 17-item Hamilton Depression Rating Scale; ICD-11, International Classification of Diseases, 11th Revision; PSD, post-stroke depression.

### Demographic and socioeconomic factors

In univariable analyses, employment status was associated with PSD (*χ*^2^ = 12.36; *p* = 0.006; Cramér’s *V* = 0.175), whereas age, sex, marital status, educational attainment, and socioeconomic status were not significantly associated. Individual vascular risk factors, including hypertension, diabetes mellitus, dyslipidemia, current smoking, atrial fibrillation, and previous stroke or TIA, were also not significantly associated with PSD (Table 1).

### Stroke characteristics associated with post-stroke depression

The distribution of stroke subtypes differed between participants with and without PSD (*χ*^2^ = 21.34; *p* < 0.001; Cramér’s *V* = 0.230). Although ischemic stroke predominated in both groups, ICH and CVST were proportionally more frequent among participants with PSD, whereas TIA was more frequent among those without PSD.

Among participants included in the binary lesion-classification analysis, cortical involvement was more frequent in those with PSD than in those without PSD (60.1% vs. 46.3%; *χ*^2^ = 7.42; *p* = 0.006; *Φ* = 0.136). Four participants with combined cortical and subcortical involvement were excluded from this binary comparison.

Among participants with ischemic stroke or TIA and an available etiological classification, large-artery atherosclerosis and cardioembolism were more frequent among those with PSD, whereas small-vessel occlusion was more frequent among those without PSD (*χ*^2^ = 47.93; *p* < 0.001; Cramér’s *V* = 0.370).

Neurological severity and functional disability demonstrated the strongest univariable associations with PSD. Participants with PSD had higher NIHSS scores than those without PSD [median (IQR), 8 (4–12) vs. 2 (1–4); Mann–Whitney *U* = 6,121; *p* < 0.001] and greater functional disability at discharge [*χ*^2^ = 161.94; *p* < 0.001; Cramér’s *V* = 0.634] (Table 3, Supplementary Figure S1).

**Table 3:** Stroke-related, treatment, and functional characteristics according to post-stroke depression status.

Variable	PSD (*n* = 172)	No PSD (*n* = 231)	Test statistic	*p* value	Effect size
A. Stroke-related characteristics and acute treatment					
Stroke subtype^a^			*χ*^2^ = 21.34	<0.001	*V* = 0.230
Ischemic stroke	138 (80.7)	185 (80.1)			
Intracerebral hemorrhage	24 (14.0)	15 (6.5)			
Cerebral venous sinus thrombosis	7 (4.1)	5 (2.2)			
Transient ischemic attack	2 (1.2)	26 (11.3)			
Cortical involvement^b^			*χ*^2^ = 7.42	0.006	*Φ* = 0.136
Cortical	101 (60.1)	107 (46.3)			
Subcortical	67 (39.9)	124 (53.7)			
TOAST etiology among ischemic stroke/TIA^c^	*n* = 139	*n* = 211	*χ*^2^ = 47.93	<0.001	*V* = 0.370
Large-artery atherosclerosis	65 (46.8)	50 (23.7)			
Cardioembolism	42 (30.2)	33 (15.6)			
Small-vessel occlusion	25 (18.0)	98 (46.4)			
Other determined etiology	4 (2.9)	19 (9.0)			
Cryptogenic	3 (2.2)	11 (5.2)			
Hyperacute reperfusion therapy^d^	41 (23.8)	41 (17.7)	*χ*^2^ = 2.25	0.133	*Φ* = 0.075
B. Neurological severity and functional status					
Functional disability at discharge (mRS)^e^			*χ*^2^ = 161.94	<0.001	*V* = 0.634
No symptoms (mRS 0)	2 (1.2)	75 (32.5)			
No significant disability (mRS 1)	26 (15.1)	89 (38.5)			
Slight disability (mRS 2)	21 (12.2)	37 (16.0)			
Moderate disability (mRS 3)	58 (33.7)	20 (8.7)			
Moderately severe disability (mRS 4)	47 (27.3)	8 (3.5)			
Severe disability (mRS 5)	18 (10.5)	2 (0.9)			
NIHSS score, median (IQR)	8 (4–12)	2 (1–4)	*U* = 6,121	<0.001	—

a Stroke-subtype classification was available for 402 participants (171 with PSD and 231 without PSD). One participant with a mixed ischemic-stroke and intracerebral-hemorrhage classification was excluded from the four-category comparison. Percentages were calculated using the corresponding available-case denominators. Values are presented as *n* (%) unless otherwise indicated.

b The cortical-versus-subcortical analysis included 399 participants (168 with PSD and 231 without PSD). Four participants with combined cortical and subcortical involvement were excluded from the binary comparison. Percentages were calculated using the corresponding available-case denominators.

c TOAST classification was considered applicable to 351 participants with ischemic stroke or TIA after excluding participants with intracerebral hemorrhage (*n* = 39) or CVST (*n* = 12). Of these, 350 had a single applicable etiological classification and were included in the analysis (139 with PSD and 211 without PSD). One participant with combined large-artery atherosclerotic and cardioembolic classifications was excluded. Percentages were calculated using the corresponding available-case denominators.

d Hyperacute reperfusion therapy comprised intravenous thrombolysis, endovascular thrombectomy, or both. Treatment data were available for all 403 participants.

e Discharge mRS and NIHSS data were available for all 403 participants. One erroneous mRS score of 6 was corrected to 5 after verification against the source record. NIHSS scores are presented as median (IQR) and were compared using the Mann–Whitney *U* test because their distribution was non-normal.

CVST, cerebral venous sinus thrombosis; mRS, modified Rankin Scale; NIHSS, National Institutes of Health Stroke Scale; PSD, post-stroke depression; SD, standard deviation; TIA, transient ischemic attack; TOAST, Trial of Org 10,172 in Acute Stroke Treatment.

### Clinical characteristics associated with post-stroke depression

Urinary incontinence, dysphagia, speech or language impairment, and motor weakness were significantly more frequent among participants with PSD than among those without PSD (all *p* < 0.001; Table 4). Mild cognitive impairment was not significantly associated with PSD (4.1% vs. 3.5%; *p* = 0.750). Hyperacute reperfusion therapy, comprising intravenous thrombolysis, endovascular thrombectomy, or both, was numerically more frequent among participants with PSD than among those without PSD (23.8% vs. 17.7%); however, this difference was not statistically significant (*p* = 0.133).

**Table 4:** Clinical neurological manifestations according to post-stroke depression status.

Variable	PSD (*n* = 172), *n* (%)	No PSD (*n* = 231), *n* (%)	Test statistic	*p* value	Effect size
Urinary incontinence	34 (19.8)	9 (3.9)	*χ*^2^ = 26.06	<0.001	*Φ* = 0.254
Dysphagia	69 (40.1)	21 (9.1)	*χ*^2^ = 54.71	<0.001	*Φ* = 0.368
Speech or language deficit	84 (48.8)	67 (29.0)	*χ*^2^ = 16.55	<0.001	*Φ* = 0.203
Mild cognitive impairment^a^	7 (4.1)	8 (3.5)	*χ*^2^ = 0.10	0.750	*Φ* = 0.016
Motor weakness	141 (82.0)	153 (66.2)	*χ*^2^ = 12.38	<0.001	*Φ* = 0.175

Values are presented as *n* (%). Percentages were calculated using the corresponding PSD and no-PSD group denominators. Categorical variables were compared using Pearson’s chi-square test. The Phi coefficient is reported as the measure of effect size.

a Mild cognitive impairment was identified using code 5 in the recorded neurological-symptom field.

PSD, post-stroke depression.

Among participants with ischemic stroke or ICH, neither stroke laterality nor vascular territory was significantly associated with PSD (Supplementary Table S1). Sensory symptoms were less frequent among participants with.

PSD than among those without PSD (3.5% vs. 9.5%; *p* = 0.018), although the association was small (*Φ* = 0.117) (Supplementary Table S2). Visual disturbance, neglect, vertigo or imbalance, and other neurological symptoms were not significantly associated with PSD (Supplementary Table S2).

### Factors independently associated with post-stroke depression

The expanded final multivariable logistic regression model included 13 predictor domains and 17 regression coefficients (Table 5). The analytical sample comprised 398 participants, including 167 with PSD and 231 without PSD. Five participants with PSD were excluded because they could not be assigned to the predefined mutually exclusive model categories: one had a mixed ischemic-stroke and ICH classification, and four had combined cortical and subcortical involvement. The model therefore included approximately 9.8 PSD events per coefficient. PSD was coded as the event of interest; therefore, an aOR greater than 1 indicates increased adjusted odds of PSD. Collinearity diagnostics showed no evidence of clinically significant multicollinearity, with all VIFs <2.3.

**Table 5:** Expanded multivariable logistic regression analysis of factors associated with post-stroke depression.

Predictor	aOR (95% CI)	*p* value
Neurological severity and functional status		
NIHSS score, per 1-point increase	1.35 (1.22–1.49)	<0.001
Unfavorable functional status, mRS 3–5 vs. 0–2	6.51 (3.19–13.29)	<0.001
Treatment and demographic characteristics		
Hyperacute reperfusion therapy, yes vs. no^a^	0.69 (0.32–1.50)	0.347
Male vs. female	1.21 (0.53–2.79)	0.648
Cerebrovascular-event subtype		0.067^b^
ICH vs. ischemic stroke	0.67 (0.23–1.95)	0.461
CVST vs. ischemic stroke	5.73 (1.34–24.50)	0.018
TIA vs. ischemic stroke	0.54 (0.11–2.57)	0.442
Cortical vs. subcortical involvement	1.36 (0.75–2.45)	0.307
Employment status		0.070^b^
Unemployed vs. employed	1.06 (0.45–2.50)	0.888
Housewife vs. employed	3.18 (1.26–8.02)	0.014
Retired vs. employed	1.83 (0.81–4.18)	0.148
Clinical neurological manifestations		
Urinary incontinence, yes vs. no	0.23 (0.07–0.84)	0.025
Mild cognitive impairment, yes vs. no	1.71 (0.69–4.24)	0.243
Speech or language impairment, yes vs. no	1.13 (0.61–2.09)	0.695
Motor weakness, yes vs. no	0.60 (0.30–1.21)	0.154
Sensory symptoms, yes vs. no	0.59 (0.15–2.34)	0.456
Dysphagia, yes vs. no	1.49 (0.63–3.49)	0.362
^a^Hyperacute reperfusion therapy comprised intravenous thrombolysis, endovascular thrombectomy, or both.		
^b^Overall *p* value obtained using a joint Wald test.		

Model performance	
Performance measure	Result
Model likelihood-ratio test	*χ*^2^ = 223.55; df = 17; *p* < 0.001
AUC (bootstrap 95% CI)	0.89 (0.86–0.92)
Hosmer–Lemeshow goodness-of-fit test	*χ*^2^ = 4.52; df = 8; *p* = 0.807
Sensitivity	72.5%
Specificity	90.5%
Positive predictive value	84.6%
Negative predictive value	82.0%
Overall classification accuracy	82.9%

PSD was coded as the event of interest (PSD = 1; no PSD = 0); therefore, an aOR greater than 1 indicates higher adjusted odds of PSD. Variables associated with PSD at *p* < 0.10 in univariable analyses, together with prespecified clinically relevant covariates, were entered simultaneously using forced-entry logistic regression. The model included 13 predictor domains and 17 regression coefficients, excluding the intercept. Cerebrovascular-event subtype and employment status were entered as categorical predictors; ischemic stroke and employed status were the respective reference categories. Other reference categories were favorable functional status (mRS 0–2), no hyperacute reperfusion therapy, female sex, subcortical involvement, and absence of each neurological manifestation.

The complete-case model included 398 participants (167 with PSD and 231 without PSD), corresponding to approximately 9.8 PSD events per coefficient. Five participants were excluded because they could not be assigned to the predefined mutually exclusive model categories: one had a mixed ischemic-stroke and ICH classification, and four had combined cortical and subcortical involvement. All variance inflation factors were <2.3. Classification measures were calculated using a probability cutoff of 0.50, and the apparent 95% CI for the AUC and the pointwise 95% confidence band for the ROC curve were estimated using 5,000 stratified bootstrap samples.

The CVST and housewife estimates should be interpreted cautiously because the corresponding overall categorical tests were not statistically significant. The inverse adjusted association for urinary incontinence may reflect suppression, overadjustment, or estimate instability and should not be interpreted as protective.

aOR, adjusted odds ratio; AUC, area under the receiver operating characteristic curve; CI, confidence interval; CVST, cerebral venous sinus thrombosis; ICH, intracerebral hemorrhage; mRS, modified This is a provisional file, not the final typeset article 24 Rankin Scale; NIHSS, National Institutes of Health Stroke Scale; PSD, post-stroke depression; TIA, transient ischemic attack.

Higher NIHSS score was independently associated with increased odds of PSD (aOR per 1-point increase, 1.35; 95% CI, 1.22–1.49; *p* < 0.001). Participants with unfavorable functional status at discharge (mRS 3–5) had more than sixfold higher adjusted odds of PSD than those with mRS scores of 0–2 (aOR, 6.51; 95% CI, 3.19–13.29; *p* < 0.001).

The overall association between stroke subtype and PSD did not reach statistical significance (joint Wald *p* = 0.067). Compared with ischemic stroke, the category-specific CVST coefficient indicated increased adjusted odds of PSD (aOR, 5.73; 95% CI, 1.34–24.50; *p* = 0.018). However, this estimate was imprecise because only 12 participants had CVST and should be interpreted cautiously in the context of the nonsignificant overall stroke-subtype test. ICH (aOR, 0.67; 95% CI, 0.23–1.95; *p* = 0.461) and TIA (aOR, 0.54; 95% CI, 0.11–2.57; *p* = 0.442) were not independently associated with PSD.

The overall employment-status test was not statistically significant (joint Wald *p* = 0.070). The category-specific coefficient for housewife status indicated higher adjusted odds of PSD compared with employment (aOR, 3.18; 95% CI, 1.26–8.02; *p* = 0.014). However, this finding should be interpreted cautiously because the overall employment-status test was nonsignificant. Unemployment (aOR, 1.06; 95% CI, 0.45–2.50; *p* = 0.888) and retirement (aOR, 1.83; 95% CI, 0.81–4.18; *p* = 0.148) were not independently associated with PSD.

Urinary incontinence showed an inverse adjusted association with PSD (aOR, 0.23; 95% CI, 0.07–0.84; *p* = 0.025), despite being positively associated with PSD in the univariable analysis. Because the direction of the association reversed after multivariable adjustment, this result should be interpreted cautiously. Potential explanations for this reversal, including suppression, overadjustment, and model instability, are considered in the Discussion.

Cortical involvement, dysphagia, hyperacute reperfusion therapy, sex, mild cognitive impairment, speech or language deficit, motor weakness, and sensory symptoms were not independently associated with PSD after multivariable adjustment (all *p* > 0.05).

**Figure 3:**
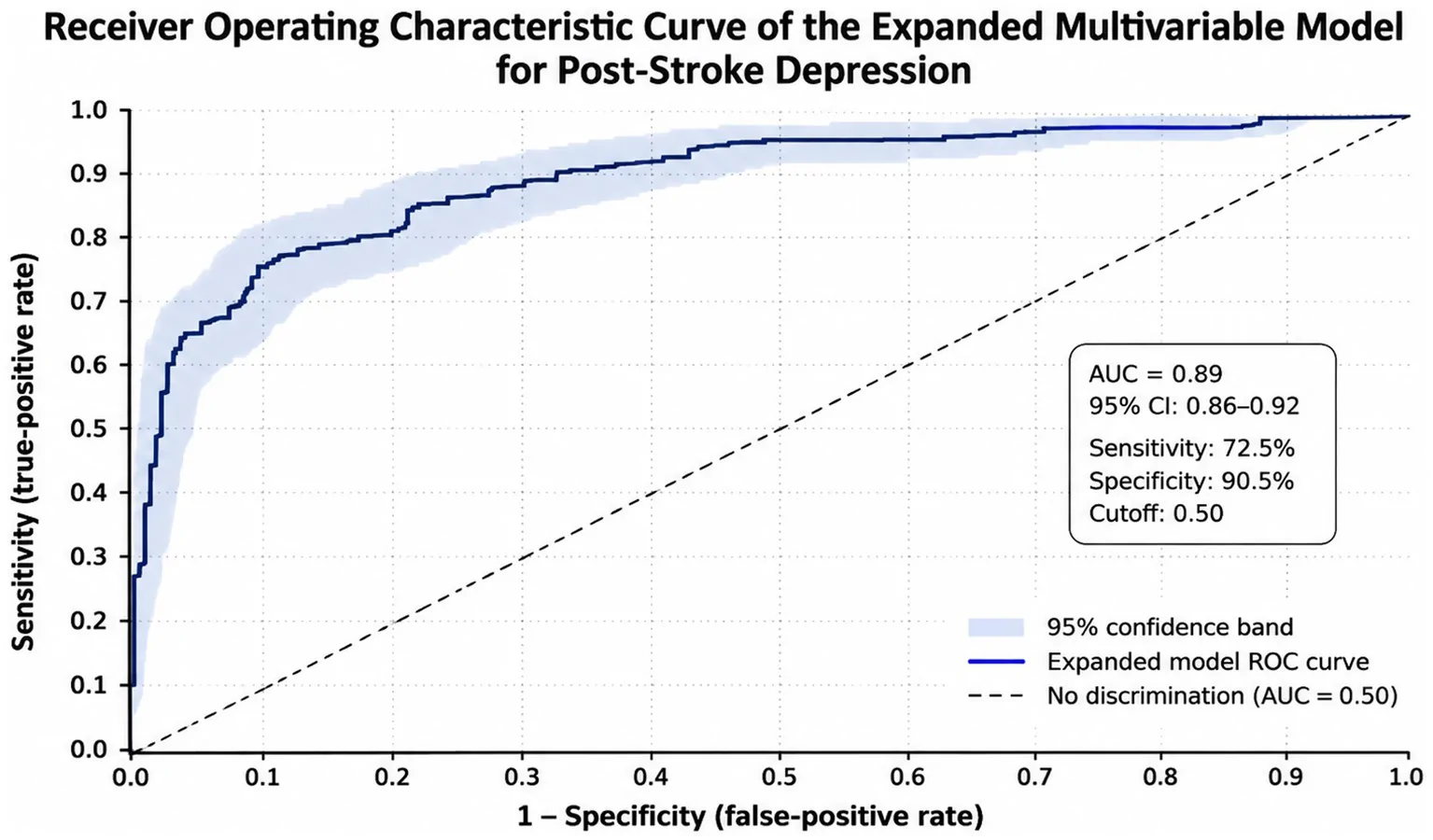
Receiver operating characteristic (ROC) curve of the expanded multivariable logistic regression model for discriminating between participants with and without post-stroke depression (PSD). The model demonstrated excellent apparent discrimination, with an area under the ROC curve (AUC) of 0.89 [bootstrap 95% confidence interval (CI), 0.86–0.92]. At the probability cutoff of 0.50, sensitivity was 72.5% and specificity was 90.5%. The solid dark-blue line represents the model ROC curve, the light-blue shaded area represents the pointwise 95% confidence band, and the diagonal dashed line represents no discrimination (AUC = 0.50). Performance was assessed in the model-development cohort and requires external validation.

**Figure 4:**
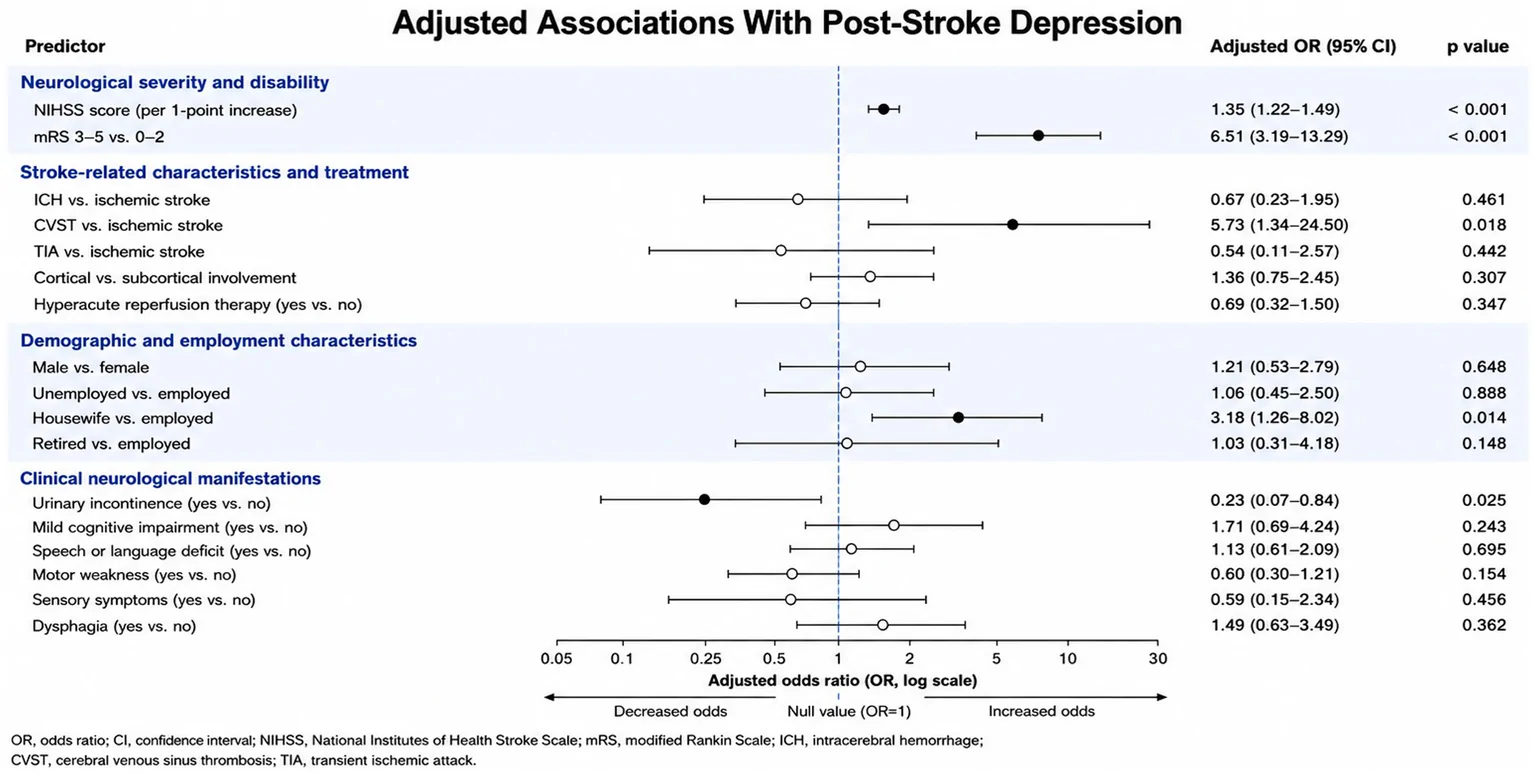
Forest plot of the expanded multivariable logistic regression model showing adjusted odds ratios (aORs) and 95% confidence intervals (CIs) for factors associated with post-stroke depression (PSD). Filled circles indicate statistically significant adjusted associations (*p* < 0.05), whereas open circles indicate nonsignificant associations. Horizontal lines represent 95% CIs, and the vertical dashed line denotes the null value (aOR = 1). Higher NIHSS scores and unfavorable functional status at discharge (mRS 3–5) were independently associated with increased odds of PSD. CVST and housewife status also showed significant category-specific associations, although the overall tests for stroke subtype and employment status were not significant. Urinary incontinence demonstrated an inverse adjusted association that should be interpreted cautiously because it may reflect suppression or overadjustment.

The final expanded model was statistically significant (likelihood-ratio *χ*^2^ = 223.55; df = 17; *p* < 0.001) and demonstrated excellent apparent discrimination (AUC, 0.89; bootstrap 95% CI, 0.86–0.92). The Hosmer–Lemeshow goodness-of-fit test showed no evidence of poor model fit (*χ*^2^ = 4.52; df = 8; *p* = 0.807). At a probability cutoff of 0.50, the model achieved a sensitivity of 72.5%, specificity of 90.5%, positive predictive value of 84.6%, negative predictive value of 82.0%, and overall classification accuracy of 82.9%. Model discrimination and adjusted associations are presented in Figures 3, 4, respectively.

## Discussion

### Prevalence of post-stroke depression

In this cohort of 403 participants with eligible cerebrovascular events, 42.7% met the diagnostic criteria for PSD. This prevalence was higher than the pooled estimate of 27% reported in a systematic review and meta-analysis, which also identified substantial heterogeneity related to study populations, assessment methods, and timing of evaluation (32).

Regional estimates have similarly varied. A Saudi study using the self-administered HADS identified depressive symptoms in 70.6% of stroke survivors, whereas an acute-stroke study from Qatar reported moderate depressive symptoms in approximately 20% of the overall cohort and 30.2% of participants from Middle Eastern or African backgrounds using the PHQ-9. An Egyptian hospital-based study using a structured clinical interview reported PSD in 36.9%, while approximately one-quarter of participants in a Jordanian prospective cohort screened positive 1 month after stroke (45–48). These estimates are not directly comparable because of differences in clinical setting, eligibility criteria, assessment timing, instruments, and diagnostic thresholds. Nevertheless, the present study adds regional evidence based on structured clinical assessment combined with the clinician-administered HDRS-17 at least 3 months after the index cerebrovascular event.

### Frequency and severity of post-stroke depression

Depressive symptoms varied in severity, although most participants with PSD had mild or moderate depression. Together, these categories accounted for 132 of 172 PSD cases (76.7%) (Figure 2). Although the clinical consequences of individual severity categories were not evaluated in this study, previous research suggests that even mild depressive symptoms may be associated with poorer rehabilitation participation, functional outcomes, and health-related quality of life (49–51). These findings support systematic depression screening across the full spectrum of symptom severity during post-stroke follow-up.

### Demographic and socioeconomic correlates

Employment status was the only sociodemographic variable associated with PSD in the univariable analysis. This finding is consistent with a recent hospital-based study in which employed stroke survivors had lower adjusted odds of PSD than those who were not employed (52). However, regional evidence is inconsistent; an Egyptian hospital-based study found no significant association between occupational status and PSD (48). Employment may be related to psychological well-being through income security, occupational identity, social participation, and perceived productivity. Conversely, neurological impairment, fatigue, cognitive difficulties, and depressive symptoms may themselves limit return to work. A systematic review similarly identified mood and fatigue as important but frequently insufficiently addressed components of post-stroke return-to-work programmes (53).

After multivariable adjustment, the overall employment-status test was not statistically significant (joint Wald *p* = 0.070). The category-specific coefficient for housewife status indicated higher adjusted odds of PSD than employment (aOR, 3.18; 95% CI, 1.26–8.02; *p* = 0.014), whereas unemployment and retirement were not independently associated with PSD. Because the overall categorical test was nonsignificant, the housewife-specific finding should be regarded as exploratory. Differences in domestic responsibilities, financial circumstances, social participation, and access to rehabilitation or psychological support are possible explanations, but these factors were not measured.

Age, sex, marital status, educational attainment, and socioeconomic status were not associated with PSD in the univariable analyses. Sex, included as a clinically relevant covariate, was also not independently associated after adjustment. Regional evidence has been inconsistent. An Egyptian study similarly found no associations with age, sex, marital status, or occupational status, although lower educational and socioeconomic status were associated with PSD (48). In contrast, women were approximately twice as likely to screen positive in a Qatari acute-stroke cohort (46), while secondary or high-school education was associated with a lower likelihood of.

PSD than no formal education in a Jordanian cohort (47). These differences may reflect variation in sample composition, cultural context, assessment timing, depression measures, and adjustment for neurological severity and disability. The predominantly male composition of the present cohort may also have limited the precision of sex-specific estimates, while marital status may not adequately represent the availability or quality of social support.

### Stroke characteristics and post-stroke depression

The distribution of cerebrovascular-event subtypes differed between participants with and without PSD. Although ischemic stroke predominated in both groups, ICH and CVST were proportionally more frequent among participants with PSD, whereas TIA was more frequent among those without PSD. The lower frequency of PSD among participants with TIA may reflect its generally lower neurological and functional burden. However, TIA was not independently associated with PSD after adjustment and should not be interpreted as protective.

The overall adjusted association between cerebrovascular-event subtype and PSD did not reach statistical significance (joint Wald *p* = 0.067). Although the category-specific CVST coefficient indicated higher adjusted odds of PSD relative to ischemic stroke (aOR, 5.73; 95% CI, 1.34–24.50; *p* = 0.018), this estimate was based on only 12 CVST cases and had a wide confidence interval. Moreover, it should be interpreted in the context of the nonsignificant overall subtype test. The CVST finding is therefore exploratory and hypothesis-generating rather than definitive. Although persistent parenchymal injury, neuroinflammation, and disruption of neural networks involved in emotional regulation have been proposed as possible contributors to neuropsychiatric manifestations after CVST, these mechanisms were not investigated in the present study (54–56).

The role of lesion location in PSD remains uncertain. Although neuroimaging studies have reported associations with lesions involving the frontal cortex, basal ganglia, thalamus, or limbic regions, reviews have not identified a single anatomical location that consistently predicts PSD across studies (21, 57). More recent lesion-symptom mapping studies suggest that different depressive symptom domains may be associated with disruption of distributed neural networks rather than a single focal lesion, supporting a network-based model of PSD (58). In the present study, broad cortical involvement was associated with PSD in univariable analysis but not after multivariable adjustment. This binary classification could not evaluate specific lesion locations, lesion volume, or network disruption. This pattern is consistent with previous reviews identifying neurological severity and physical disability as reproducible correlates of PSD while reporting less consistent evidence for lesion location (57, 59).

### Clinical symptoms and stroke-related complications

Neurological severity and functional disability demonstrated the strongest adjusted associations with PSD. Each 1-point increase in NIHSS score was associated with a 35% increase in the adjusted odds of PSD, while participants with unfavorable functional status at discharge (mRS 3–5) had more than sixfold higher adjusted odds than those with mRS scores of 0–2. The marked difference in the distribution of discharge mRS scores between the PSD and no-PSD groups is illustrated in Supplementary Figure S1. These findings are consistent with systematic reviews identifying neurological severity and physical disability as among the most reproducible correlates of PSD and with recent evidence demonstrating the predictive value of functional, physical, and cognitive assessments (5, 59). Nevertheless, these observational associations should not be interpreted as establishing causality.

Urinary incontinence, dysphagia, motor weakness, and speech or language impairment were more frequent among participants with PSD in the univariable analyses, whereas sensory symptoms demonstrated a weak inverse association. Mild cognitive impairment was not associated with PSD in either the univariable or adjusted analysis. After adjustment, dysphagia, motor weakness, mild cognitive impairment, speech or language impairment, and sensory symptoms were not independently associated with PSD. The attenuation of these.

associations may reflect their relationships with overall neurological severity, functional disability, and one another. However, the absence of adjusted statistical significance does not establish that these manifestations have no clinical or psychological relevance.

Urinary incontinence demonstrated an unexpected inverse adjusted association with PSD despite its positive univariable association. This reversal could reflect statistical suppression, overadjustment, or estimate instability arising from the close relationships among urinary incontinence, NIHSS score, functional disability, and other neurological manifestations. It should not be interpreted as evidence that urinary incontinence protects against PSD.

Speech or language impairment was associated with PSD in the univariable analysis but not after multivariable adjustment. A recent systematic review and meta-analysis estimated a pooled depression prevalence of 31.7% among people with post-stroke aphasia, confirming a substantial burden in this population (60). Because patients with severe aphasia precluding reliable psychiatric assessment were excluded from the present study, our findings should not be generalized to patients with severe communication impairment.

Although dysphagia was not independently associated with PSD in our adjusted model, a prospective ischemic-stroke registry study reported an independent association between dysphagia and depressive symptoms. Differences in the populations studied, outcome definitions, assessment timing, and covariate adjustment may account for the differing findings (61).

Hyperacute reperfusion therapy was not independently associated with PSD after adjustment. However, treatment allocation was nonrandomized and depended on cerebrovascular-event subtype, stroke severity, treatment eligibility, and time of presentation. Consequently, this finding should not be interpreted as evidence that reperfusion therapy either reduces or increases the likelihood of PSD.

Overall, NIHSS score and functional status were the only factors demonstrating strong adjusted associations, relatively precise estimates, and clear overall statistical support. By contrast, the CVST and housewife coefficients arose within categorical predictors whose overall tests were nonsignificant, while the urinary incontinence estimate reversed direction after adjustment. These findings should therefore be interpreted more cautiously than the robust associations observed for neurological severity and functional disability.

### Conceptual implications

The findings suggest that PSD was more strongly associated with neurological severity and functional dependence than with broad lesion classifications or individual neurological manifestations. This pattern is compatible with a multidimensional framework in which neurological injury, disability, psychosocial consequences, and disruption of distributed neural networks may interact in the development of depressive symptoms. However, these mechanisms were not directly investigated, and this interpretation remains conceptual. Comprehensive neurological and functional assessment may help identify patients who warrant closer psychological evaluation.

### Clinical implications

Routine PSD screening should include patients without overt or severe depressive symptoms because even mild symptoms may be clinically relevant and have been associated with poorer rehabilitation participation, functional outcomes, and health-related quality of life. Patients with greater neurological severity or functional dependence may particularly benefit from early psychological assessment, multidisciplinary rehabilitation, and closer follow-up. Integrating standardized depression screening into routine post-stroke care may facilitate earlier recognition and appropriate management of PSD.

Neurological severity and functional status may provide useful indicators of vulnerability but should not be used as substitutes for direct depression assessment. Furthermore, because the multivariable model was developed and evaluated in the same cohort, it should not be used for routine clinical risk prediction before appropriate internal and external validation.

The expanded model demonstrated excellent apparent discrimination within the study cohort (AUC, 0.89), indicating potential value for clinical risk assessment. However, the model was developed and evaluated using the same cohort, and its performance may therefore be optimistic. Internal validation with optimism correction and subsequent external validation are required before the model can be recommended for clinical prediction or routine risk stratification.

The absence of independent associations for several clinical and stroke-related factors should not be interpreted as evidence that they are unrelated to PSD. Associations involving dysphagia, cortical involvement, and other neurological manifestations may have been attenuated after adjustment for NIHSS score and functional disability because these variables reflect overlapping aspects of neurological burden. Broad anatomical classifications, exclusion of patients with severe aphasia or cognitive impairment, and small numbers in some predictor categories may also have limited the detection of certain associations.

### Strengths

This study has several strengths. First, it included a relatively large, prospectively assembled hospital-based cohort derived from routine clinical practice at a comprehensive stroke center. To our knowledge, it represents one of the larger clinician-assessed PSD cohorts reported from Saudi Arabia and the Middle East. Second, PSD assessment combined structured clinical evaluation with the clinician-administered HDRS-17 and DSM-5-TR-and ICD-11-informed diagnostic criteria rather than relying solely on a self-report screening instrument. Third, the availability of demographic, socioeconomic, neurological, functional, and clinical data enabled evaluation of a broad range of factors associated with PSD within a single analytical framework. Fourth, neurological severity and functional disability were assessed using internationally validated measures, facilitating comparison with previous studies.

The inclusion of participants with CVST is an additional strength because evidence concerning PSD in this uncommon cerebrovascular condition remains limited. The observed association provides a hypothesis-generating finding for evaluation in larger CVST-specific cohorts. Finally, the multivariable analysis included assessment of multicollinearity and model performance, while adherence to STROBE guidance strengthened reporting transparency.

### Limitations

Several limitations should be acknowledged. First, although participants were prospectively enrolled, PSD was assessed once at a follow-up visit occurring at least 3 months after the index cerebrovascular event. The present analysis was therefore cross-sectional and could not establish causality, temporal changes in depressive symptoms, or the direction of associations between PSD and functional status.

Second, this was a single-center study conducted at a tertiary comprehensive stroke center, which may limit generalizability to other healthcare settings and community-based populations. Residual confounding is also possible because potentially relevant factors—including lesion volume, detailed lesion location, rehabilitation intensity, vascular risk burden, pre-stroke social support, social isolation, and stressful life events—were not systematically available.

Third, patients with severe aphasia or severe cognitive impairment that precluded reliable psychiatric assessment were excluded. Although this approach supported diagnostic reliability, it may have introduced selection bias and resulted in underrepresentation of patients with the most severe neurological impairment.

Fourth, the CVST estimate was based on only 12 participants and had a wide confidence interval. Moreover, the overall stroke-subtype test was not statistically significant; consequently, the category-specific CVST association should be considered exploratory. The complete-case model included 167 PSD events and 17 regression coefficients, corresponding to approximately 9.8 events per coefficient. Although close to the commonly used threshold of 10, sparse data in individual categories and the number of included coefficients may have produced unstable estimates or overfitting.

Fifth, model performance was assessed in the same cohort used to develop the model. The bootstrap procedure estimated the confidence interval for the AUC but did not provide full internal validation or correct the model’s performance for optimism. The reported discrimination and classification estimates may therefore be optimistic and require internal and external validation.

Finally, HDRS-17 assessments were conducted by trained neurology residents and reviewed by a stroke neurologist; however, assessors were not formally blinded to neurological severity or functional status, and formal inter-rater reliability was not evaluated. These factors may have introduced observer bias.

Future multicenter longitudinal studies incorporating repeated psychiatric assessments, detailed neuroimaging, psychosocial and rehabilitation-related factors, blinded outcome assessment, and external model validation are needed to confirm these findings and clarify the temporal course of PSD.

## Conclusion

PSD affected approximately two-fifths of participants and was independently associated with greater neurological severity and functional disability. Although the category-specific CVST coefficient indicated higher adjusted odds of PSD, the overall stroke-subtype test was nonsignificant, the number of CVST cases was small, and the estimate was imprecise; this finding should therefore be considered exploratory. Most individual neurological manifestations and stroke-related characteristics were not independently associated with PSD after adjustment. These findings support integrating standardized depression screening into comprehensive post-stroke care, particularly for patients with greater neurological impairment or functional dependence.

## Data Availability

The raw data supporting the conclusions of this article will be made available by the authors, without undue reservation.
