# Supplementary material for: Prevalence and factors associated with post-stroke depression: a cross-sectional analysis of a prospectively assembled cohort in Eastern Saudi Arabia
Source: Front Neurol. 2026 Aug 26;17:1863484. doi: 10.3389/fneur.2026.1863484 (PMC13553280; doi:10.3389/fneur.2026.1863484)
Supplement: Supplementary file 1 [file Supplementary_file_1.docx]

**Supplementary Table S1. Additional Lesion Characteristics Among Participants With Ischemic Stroke or Intracerebral Hemorrhage**

| **Variable** | **PSD (n=162), n (%)** | **No PSD (n=200), n (%)** | **Test statistic** | **p value** | **Effect size** |
| --- | --- | --- | --- | --- | --- |
| Stroke laterality¹ |  |  | Fisher–Freeman–Halton exact test | 0.174 | V=0.103 |
| Right | 79 (48.8) | 103 (51.5) |  |  |  |
| Left | 80 (49.4) | 97 (48.5) |  |  |  |
| Bilateral | 3 (1.9) | 0 (0.0) |  |  |  |
| Vascular territory² | n=158 | n=200 | χ²=0.01 | 0.917 | Φ=0.006 |
| Anterior circulation | 109 (69.0) | 139 (69.5) |  |  |  |
| Posterior circulation | 49 (31.0) | 61 (30.5) |  |  |  |

**Footnotes:**

¹ Lesion-laterality analysis was restricted to 362 participants with ischemic stroke or ICH (162 with PSD and 200 without PSD). Participants with TIA or CVST were excluded. Codes 1 and 3 represented right-sided involvement, codes 2 and 4 represented left-sided involvement, and the 1+2 code represented bilateral involvement. The Fisher–Freeman–Halton exact test was used because of the small bilateral cell counts.

² The anterior-versus-posterior circulation analysis included 358 participants with ischemic stroke or ICH (158 with PSD and 200 without PSD). Four participants with combined anterior- and posterior-circulation involvement were excluded from the binary comparison. Percentages were calculated using the corresponding available-case denominators.

Cramér’s V is reported for stroke laterality, and the Phi coefficient is reported for vascular territory.

**Abbreviations:** CVST, cerebral venous sinus thrombosis; ICH, intracerebral hemorrhage; PSD, post-stroke depression; TIA, transient ischemic attack.

**Supplementary Table S2. Additional Neurological Manifestations According to Post-Stroke Depression Status**

| **Variable** | **PSD (n=172), n (%)** | **No PSD (n=231), n (%)** | **Test statistic** | **p value** | **Effect size** |
| --- | --- | --- | --- | --- | --- |
| Sensory symptoms, including pain or sensory loss | 6 (3.5) | 22 (9.5) | χ²=5.55 | 0.018 | Φ=0.117 |
| Visual disturbance | 17 (9.9) | 23 (10.0) | χ²<0.01 | 0.981 | Φ=0.001 |
| Vertigo or imbalance | 21 (12.2) | 20 (8.7) | χ²=1.36 | 0.243 | Φ=0.058 |
| Other neurological symptoms | 0 (0.0) | 4 (1.7) | Fisher’s exact test | 0.139 | — |

**Footnote**:

Values are presented as n (%). Percentages were calculated using the corresponding PSD and no-PSD group denominators. Pearson’s chi-square test was used unless otherwise specified. Fisher’s exact test was used for other neurological symptoms because of the small expected cell counts. Sensory symptoms met the univariable model-entry criterion and were included in the expanded multivariable model but were not independently associated with PSD after adjustment.

Abbreviations: PSD, post-stroke depression.

**Supplementary Figure S1.** Distribution of functional disability at discharge according to post-stroke depression status. The stacked bars show the within-group distributions of modified Rankin Scale (mRS) scores among participants with PSD (n=172) and without PSD (n=231). Participants with PSD had substantially greater functional disability at discharge than those without PSD (χ²=161.94; p<0.001; Cramér’s V=0.634). Percentages may not total exactly 100% because of rounding. PSD, post-stroke depression.
